# The Eradication of Mongrelism

**Published:** 1897-09

**Authors:** C. F. Menninger

**Affiliations:** Topeka, Kan.


### THE ERADICATION OF MONGRELISM.

C. F. MENNINGER, M. D., TOPEKA, KAN.

The practice of medicine by homœopathic physicians that does not accord with the teachings of Samuel Hahnemann, as set forth in *The Organon, Chronic Diseases*, and his lesser writings, or with the practice of such of his ablest disciples, as Constantine Hering and Carroll Dunham, deserves to be branded as mongrelism. This kind of practice manifests itself in many ways, but chiefly in *the prescription, the manner of arriving at it*, and *its composition.* Could these be rectified, so as to accord with the teachings and practice of Hahnemann, Hering, and Dunham, there would be little left needing alteration, and this can be reached in but one way, namely: by education.

We may sum up the evils that are combating the principles of true Homœopathy, in the Prescription on the Diagnosis, and Polypharmacy. Under the former we would include prescribing based upon the diagnosis of the disease, as well as that made upon a pathological basis. No greater mistakes are made by homœopathic physicians than in attempting to imitate the allopaths. Far too great a number of homœopaths examine a patient with a view to arriving at a diagnosis, and, resting there, prescribe. Their prescription is a mere routinism; in every case of pneumonia they give Bryonia and Phosphorus, and in intermittent fever, Arsenicum and China. And so on, for the entire catalogue of diseases, they have some one or more remedies at hand as soon as diagnosed. Here the strict inductive method of Hahnemann has been lost sight of and nothing but failure, ignominious failure will be the harvest.

Blinded by theoretical images of diseased tissues, the pathological prescriber reaps the same results. That kaleidoscopic science is very attractive and allures the physician into the net so deftly that he scarcely realizes the injuries he has inflicted. He has no misgivings that his view is erroneous. Yet the history of medicine shows that the time will arrive when he must surely discard the theory which he has trusted so implicitly and set up another in its stead. Certainly we will not be surprised that much error must prevail, much damage be done, and much failure be experienced when we reflect that so much medical treatment depends upon untrustworthy *views* of the nature of the diseased state, and upon deductions as to treatment which, by reason of being dependent upon these views, must be still more unreliable, and even dangerous. If the pathologist be governed by facts, he, more than the homœopathist, deserves the term which he has made invidious—"Symptom Coverer." If the patient have a high fever, the pathologist does not hesitate to immerse the sufferer's whole body in a bath of ice-cold water, and that, too, when dealing with such fell diseases as pneumonia and typhoid fever. If the bowel be sluggish, it is urged by a purgative. If it be too lax, an agent that will practically plug it up is administered, and so on through the list. Remedies thus given for individual symptoms are selected without any regard to their influence upon the other symptoms in the case, and as each symptom thus has its own therapeutic measure, a wondrous structure of polypharmacy is reared, where the drugs are combined, not with regard to their pathogenetic effects, but with reference to their chemical reaction upon each other.

The view here given does not, we believe, do injustice to the old school, and, reluctantly, we acknowledge that it fits many in our own camp, who do not appreciate, nay, who do not know, that pearl of great price, the method of Hahnemann. They can hardly be appreciative of it, else they would not imitate the old-school pathology. They would not prescribe, as do the members of the old school, massive doses of drugs on pathological conclusions. They would not show the contempt they do for strict homœopathists, nor so persistently ignore their teachings and example.

### POLYPHARMACY.

Truly our friends give us more trouble and annoyance than our enemies. And in no respect are they threatening to take our very life more certainly than in their practice of polypharmacy. Mongrelism here reaches its highest and most prevalent type of development. It has become rampant, developing into a frightful epidemic, by reason of the astonishingly careless and inadequate instruction in our colleges and shocking cupidity and selfishness of our pharmacists. Nothing has done more to retard the progress of Homœopathy than polypharmacy. Nothing has done more to injure the prospects of Homœopathy for the future than polypharmacy. Nothing will more surely and effectually kill Homœopathy than polypharmacy. By this term, polypharmacy, we mean any mixture of remedial agents in the treatment of disease, and consequently alternation of rotation, whether of the same or different potencies as well as that monstrosity of homœopathic sugar pill—the compound tablets—are included. If we turn to *The Organon* we see that Hahnemann says:

"In the treatment of disease, only one *simple* medicinal substance should be used at a time."

This does not mean that if one remedy does not cover the entire symptom picture we are to add to that another one that will cover the remainder. No possibility of giving two remedies according to that section. Nor does he leave us in doubt, for he says:

"It is impossible to conceive why there should be the least doubt as to whether it is more natural and rational to prescribe a single well-known medicine at a time for a disease, or to give a mixture composed of several different medicines.

"Perfectly simple, unmixed, and simple remedies afford the physician all the advantages he could possibly desire. He is enabled to cure natural diseases safely and permanently, through the homœopathic affinity of those artificial morbific potencies; and in obedience to the wise maxim that 'it is useless to apply a multiplicity of means, where simplicity will accomplish the end,' he will never think of giving more than one simple medicine at a time. Even in taking it for granted that all simple medicines were completely proved with regard to their pure and peculiar action upon the healthy human body, the physician would abstain from mixing and compounding drugs, aware that it is impossible to foresee the variety of effects that two or more medicines contained in a mixture might have; or how one might modify and counteract the effect of the other, when introduced into the human body. It is equally certain, on the other hand, that a simple medicine, well selected, will by itself be quite sufficient to give relief in diseases whereof the totality of symptoms is accurately known. Supposing, even, that a medicine had not been selected quite in accordance with the similitude of symptoms, and that, consequently, it did not alleviate the disease, it would nevertheless be useful by adding to our knowledge of curative remedies. By calling forth new symptoms in such a case, the medicine might corroborate those symptoms which it had already manifested in experiments upon healthy persons—an advantage which is not to be gained by the use of compound medicines."

How is it that, in the face of such explicit directions from the founder of Homœopathy, so many have become infected with this blighting innovation? The only answer to this can be that on their part there must be a lack of knowledge, coupled with evil influences that have co-operated to bring about this condition, or that the founder of Homœopathy was wrong in his declarations. This latter supposition is clearly untenable, and cannot bear the scrutiny of honest investigators, because there are and have been strict followers of Hahnemann who, being taught polypharmacy in old-school colleges, practiced it until, in confusion worse confounded, they abandoned it; and on reading, and becoming thoroughly posted in the works of Hahnemann and the practice of his closest disciples, have adopted his instructions, and, following them to the letter, have achieved the most marvelous results, as did Hahnemann himself; far better than could possibly have been attained in any other way. This kind of practice has become the beacon light that has, and we say *must*, lead the steady and triumphant progress of Homœopathy's hosts. Blot out this beacon light, and the inevitable consequence will be ruin. "Any homœopathic physician who allows himself to drift into polypharmacy, from that moment drifts away from Homœopathy." Then why have so many drifted away from Homœopathy into mongrelism? The only answer then can be *Ignorance* and *Evil Influences*.

Before taking up the subject of polypharmacy let me express my views on the relation of the pharmacists to it. Have they been some of the evil influences at work? To this I have not the slightest hesitancy to say YES. The fault is not ALL with the pharmacists. Yet they have and are exerting a most baneful influence in this matter, one that will work evil to all concerned. They have and are preparing combinations of homœopathic medicines in almost inconceivable numbers. They have and are still continuing to have the temerity to ask homœopathic physicians to buy and use them. They have and still continue to have the audacity to send traveling salesmen and saleswomen to all parts of the United States, who boldly tell of their sales, and unhesitatingly and persistently urge homœopathic physicians to buy them and use them regardless of all proper indications. "These pharmacy venders unblushingly attempt to tell us exactly what these combinations will cure, and thus insinuatingly encourage in the unwary doctor a lazy, uncertain, and fruitless system of practice." In this way they, by their persistency, virtually force upon the profession all sorts of compounds. The plea of the pharmacists that they must meet the demands of their customers has something of truth in it, but it is not all the truth. Polypharmacy arose, not from an original demand of the profession, but in reality originated with a few unscrupulous manufacturers who have gradually, by various methods, induced a certain portion of the profession to use their unauthorized preparations. In this way a demand has been created, and some physicians beguiled into encouraging this false system. Why this sort of pharmaceutical quackery has been so long tolerated by the colleges and the profession is difficult to understand. Nothing that has yet been done by its avowed enemies is so surely calculated to retard the steady and triumphant progress of Homœopathy as this insidious work of pharmacists. There has been an unexpressed feeling prevalent that this evil would soon be corrected through the silent protest and lack of patronage of the better class of homœopathic physicians. It was a false hope, and the time has come when some decided and unequivocal action should be taken by the profession at large, by our colleges, and by our teachers. Our society, though yet in its infancy, must meet this question without hesitancy or compromise. We must at once take active measures against this evil and place ourselves squarely for Homœopathy and not against it.

This question of polypharmacy, etc., which we think is the soul and body of mongrelism, has been so frequently brought before the profession, and in such various ways, that there scarcely remains a single one who has not heard of it in all the length and breadth of this land. And yet we would maintain that the chief factor of causation is *Ignorance.* Ignorance on the part of the profession, not of the sections of *The Organon*, heretofore quoted in full, but an ignorance of *how to do better than to prescribe the compound tablet*. Through his ignorance the practitioner loses all faith and confidence in his single dynamized remedy and necessarily drifts into the grossest materialism and polypharmacy. Homœopathy appeals only to the minds of the educated. The ignorant know nothing of it and will have none of it. But if the practitioner and student of Homœopathy only knew the better way—knew how to prescribe the single dynamized remedy; knew it so well and so thoroughly that he could tell another how it must be done; if he could but once after knowing it thoroughly, realize by practice the immense superiority of it, he would never again resort to polypharmacy. He would really have faith and increasing confidence in his remedy, his successes would be marked, and there would come to him a satisfying conviction that he was making real progress in a difficult art. The consequences following necessarily in the train of this kind of practice would be of the utmost worth to him and to Homœopathy in its onward progress and triumph.

But the question now remains, "Why is a *homœopathic physician ignorant of the very essence of his business? Because it has never been taught him.* He has been taught everything else, but not this one thing: "*The Philosophy of Homœopathy and The Art of applying it.*" Only within the last few years has there been the opportunity for a student of Homœopathy to learn this in the medical colleges. Those who learned it prior to that time did so through some of the worst of failures. True, they had heard of it in the colleges, but only in so haphazard and often sneering a manner that it made anything but a good impression. After many failures, the undaunted student of Homœopathy laboriously climbed to the heights that gave him an unobstructed view. There are but few who have attained this eminence, and it is to no one's credit but their own. It is high time now that our medical colleges take up this work. It belongs to them, and the profession must demand that they put forth every effort to thoroughly ground the student in all things appertaining to Homœopathy. In how many homœopathic medical colleges in this country do we find *The Organon* taught as one of the leading branches? Until within the last few years there was not a single one. Now at this present day there is one and only one. We will never have homœopaths like Dunham, Hering, Bœnninghausen, Lippe, and Jahr until *The Organon*, *Chronic Diseases*, and *Lesser Writings* of Samuel Hahnemann are studied as among the principal subjects in our medical colleges. It must not be taught by a student teacher. It must be assigned to the ablest man in the faculty, and he must have the co-operation of every other member of that body. It must not be a secondary study; instead, it must be and is of the utmost importance, of primary worth, and must be taught every day of every session throughout the entire time of four years. The student cannot become too proficient in this knowledge. Nor would I limit the instruction to Hahnemann's writings alone. The classic works of Dunham, the exposition of homœopathic philosophy, and the art of its practice by Hering, Lippe, Hemple, Rau, Goullon, and a host of others should all be included under this subject.

When a student has had a four-years' course as above outlined in addition to all the other branches of medicine and surgery as they are now taught in our medical colleges, no enemy of Homœopathy can do him harm. He will be impregnable against any and all assaults.

What is our duty in this matter? is the question that finally comes to us for answer. As students and practitioners of Homœopathy, individually and collectively, we must demand this of all of our colleges. Our local, State, Interstate, and National societies must take steps at once to bring this before the colleges in such terms as to command compliance. Our society must demand of the Inter-Collegiate Committee of the American Institute of Homœopathy, that they require this of all of our colleges. These and all other active measures must be taken *now* to rid us of this leviathan of mongrelism that is momentarily taking our life-blood. We must stand united now and forever for Homœopathy, pure and simple, and all that the term implies.—*The American Homœopathist, May 15th, 1897.*

